# Mechanisms underlying of antiretroviral drugs in different cellular reservoirs with a focus on macrophages

**DOI:** 10.1080/21505594.2020.1760443

**Published:** 2020-05-06

**Authors:** Stefano Aquaro, Ana Borrajo, Michele Pellegrino, Valentina Svicher

**Affiliations:** a Department of Pharmacy, Health and Nutritional Sciences, University of Calabria, Rende, Italy; b Department of Experimental Medicine, University of Rome Tor Vergata, Roma, Italy; c Department of Microbiology and Parasitology, Faculty of Pharmacy, Complutense University of Madrid, Madrid, Spain

**Keywords:** HIV-1, macrophages, virus reservoir, antiretroviral drugs target

## Abstract

Ongoing with current combinations of antiretroviral drugs for the treatment of Human Immunodeficiency Virus (HIV) infection can successfully maintain long-term suppression of HIV-1 replication in plasma. Still, none of these therapies is capable of extinguishing the virus from the long-lived cellular reservoir, including monocyte-derived macrophages (MDM), that means the principal obstacle to HIV cure. MDM are widely distributed in all tissues and organs, including central system nervous (CNS) where they represent the most frequent HIV-infected cells that means the principal obstacle to HIV cure. Current FDA-approved antiretroviral drugs target viral reverse transcriptase, protease, integrase, and entry processes (coreceptor or fusion blockade). It is desirable to continue to develop new antiretrovirals directed against alternative targets in the virus lifecycle in order to further optimize therapeutic options, overcome resistance to existing medications, and potentially contribute to the elimination of viral reservoirs.

This review provides a comprehensive overview of the activity of antiretroviral drugs (classical and upcoming) in monocytes-derived macrophages (MDM). Defining the antiviral activity of these drugs in this important cellular HIV-1 reservoir provides crucial hints about their efficacy in HIV-1 infected patients.

## Introduction

The AIDS-related mortality has dropped sharply, and AIDS has gradually become a controllable, chronic disease. Thanks to a highly effective antiretroviral therapy. Based on global AIDS response progress report, there are nearly 17 million people receiving antiretroviral therapy in 2016 [1] and this number could reach 24 million by 2020.

The development of drugs for human immunodeficiency virus (HIV) infection began soon after the virus was discovered 25 years ago [2]. When used daily as prescribed, combined antiretroviral therapy (cART) (usually based on the administration of three drugs) suppresses HIV replication and thus limits disease progression. As a consequence, HIV-infected patients are now expected to live to an old age as long as an oral HIV drug regimen can be continued. Thus, cART has transformed HIV from a terminal illness to a chronic disease [3].

Despite this success, the eradication of HIV from the body is not achievable, and the main reason is the presence of virus reservoirs. Monocyte-derived macrophages (MDM) are one of the major cellular targets for HIV-1 infection and an important virus reservoir. MDM contribute to the transmission and the pathogenesis of HIV-1 infection throughout the progression of HIV-1 infection, especially at late stages when CD4⫹ T lymphocytes have been depleted extensively [4–8].

HIV-infected MDM are frequently found in the blood and generally distributed in all tissues, organ, and compartments [7,8]. In the central nervous system (CNS), MDM and microglia cells represent the most common cell lineages that support virus replication, thus being responsible for the onset of HIV-associated dementia and the neuropathological features of HIV encephalitis [7–10].

Tissue macrophages are critical contributors to HIV pathogenesis, however, their specific role in HIV persistence during long-term suppressive ART has been demonstrated by Honeycutt et al. [11]. In this work, using humanized myeloid-only mice, it was shown that HIV infection of tissue macrophages is rapidly suppressed by ART, as reflected by a rapid drop in plasma viral load and a dramatic decrease in the levels of cell-associated viral RNA and DNA. No viral rebound was observed in the plasma of the ART-treated animals at 7 weeks after ART interruption, and no replication-competent virus was rescued from the tissue macrophages obtained from these animals. In contrast, in a subset of animals, a delayed viral rebound was observed that is consistent with the establishment of persistent infection in tissue macrophages. These observations represent the first direct evidence, to our knowledge, of HIV persistence in tissue macrophages *in vivo* [11].

On this basis, we reviewed the activity of antiviral compounds of clinical interest, as well as the factors affecting their efficacy. The current studies consider the relevance of knowing new therapeutic strategies able to prevent HIV-1 replication in MDM, focusing also the attention on current and original compounds. Thus, new and innovative anti-HIV-1 therapeutic approaches directed to HIV-1-infected MDM are briefly described.

## Cellular HIV-1 reservoirs and HIV latency in monocytes/macrophages

Monocytes are bone marrow-derived mononuclear phagocyte cells that circulate in the blood for few hours/days before being recruited into tissues [9,10,12]. The expression of various chemokine receptors and cell adhesion molecules at their surface allows them to exit the bone marrow into the blood and to be subsequently recruited from the blood into tissues [9,11].

Monocytes represent approximately 10% of leukocytes in the human peripheral blood, with a considerable pool located in the spleen and lungs, as well as homing into inflammatory sites in response to specific chemokines [10]. Monocytes belong to the innate arm of the immune system providing responses against viral, bacterial, fungal, or parasitic infections [9,13]. Their functions include the killing of pathogens via phagocytosis, the production of reactive oxygen species (ROS), nitric oxide (NO), myeloperoxidase, and inflammatory cytokines [13]. Under specific conditions, monocytes can stimulate or inhibit T-cell responses during cancer as well as infectious and autoimmune diseases. They are also involved in tissue repair and neovascularization [14].

The failure of cART in eradicating HIV infection has underlined the relevance of the presence of HIV-1 reservoirs in the body. HIV-1 can evade immune response by several mechanisms, including the establishment of persistent infection within different cell types, including memory or naive T lymphocytes and MDM. In particular, MDM represent an important HIV-1 cellular reservoir as they can survive to HIV-1 cytopathic effect for prolonged periods of time (particularly microglia or alveolar macrophages) [15–22], thus allowing HIV-1 spreading into anatomical sanctuaries.

Studies demonstrated that HIV-1 can be detected in circulating monocytes from patients on cART for prolonged periods of time [23–25]. Interestingly, these monocytes had produced undetectable amounts of HIV-1 RNA under basal conditions, but the virus can reactivate following appropriate stimulation [23,25].

Another key feature of macrophages is represented by their capability to spread the virus to CD4 + T cells. MDM HIV-infected have been shown to fuse with autologous and heterologous CD4 + T cells thus allowing HIV-1 transmission to these cells [25–27].

HIV-1 replication in macrophages is regulated by cytokines and other extracellular stimuli. Based on the stimuli or cytokine profile, macrophages can be polarized into either M1 (classically activated) or M2 (alternatively activated) [28,29]. Cassol and colleagues reported that M1/M2 polarization of MDMs was associated with poor CCR5-dependent HIV-1 infection as compared to non-polarized MDMs.

## Historical background of antiretroviral therapy

**Figure 1.**
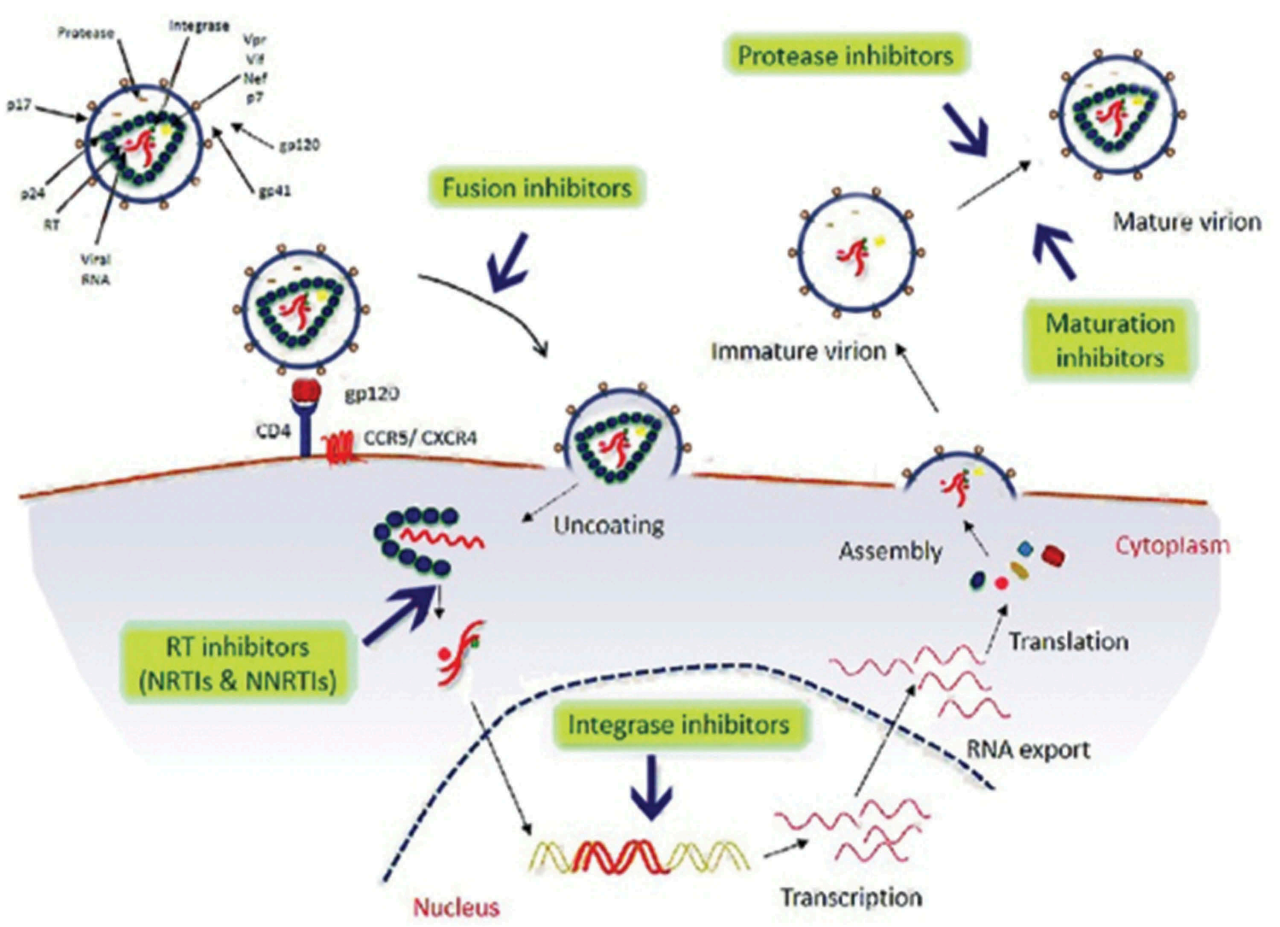
Schematic representation of steps of HIV-1 life cycle and targets of the currently available antiretroviral drugs.

Antiretroviral drugs act by interfering with vital viral replication processes and are classified according to the step they inhibit in the viral life-cycle (Figure 1). A sub-classification may be based on their chemical structure. A milestone in the history of HIV disease has been the availability of new classes of drugs, in 1995–96, allowing the introduction of combination ARV therapy (HAART) and the gradual evolution of HIV infection into a chronical, usually nonfatal condition [30,31]. Currently, there are seven categories of ARV drugs: Nucleoside Reverse Transcriptase Inhibitors (NRTIs), Non-Nucleoside Reverse Transcriptase inhibitors (NNRTIs), Protease Inhibitors (PIs), drugs that interfere with viral entry (Fusion Inhibitors [FI] and CCR5 antagonists like maraviroc), Integrase Inhibitors (INIs) and Maturation Inhibitors (MI, in late-stage clinical trial).

Also, Integrase strand transfer inhibitors (INSTIs) are the class of ARV drugs most recently approved by the FDA for the treatment of HIV-1 infections. INSTIs block the strand transfer reaction catalyzed by HIV-1 integrase and have been shown to potently inhibit infection by wild-type HIV-1. The new INSTIs, Bictegravir (BIC), are currently in late-stage clinical trial [31].

There is an important topic about the ARV drug tissue penetration. In a recent study, it was shown that for many commonly used ARVs, drug penetration was lower in lymphoid tissue cells than that observed in blood cells [32]. These findings – that measuring drug concentrations in plasma or in PBMCs does not predict those in lymphoid compartments where most viral replication actually occurs and that viral replication persists in the lymphatic tissue of some patients – provide a compelling case and rationale to develop new ART strategies that will fully suppress virus production at its source [32]. In this way, the long-term consequences of persistent virus production for reservoir replenishment and tissue pathologies that restrict immune reconstitution can be averted, and the foundations can be laid for a potential functional cure for HIV-1 infection.

Because many of antiretroviral drugs belong to the same drug class, if HIV becomes resistant to one drug in a class, it may have varying degrees of resistance to other drugs in the same family. This is called cross-resistance and it can limit future treatment options. Cross-resistance highlights the need for new effective therapies.

## Inhibitors that target the retroviral enzyme reverse transcriptase (RT)

Inhibitors that target the HIV-1 reverse transcriptase (RT) enzyme hav played an indispensable role in the treatment and prevention of HIV-1 infection. They can be grouped into two distinct therapeutic groups, namely the nucleoside and nucleotide RT inhibitors (NRTIs), and the non-nucleoside RT inhibitors (NNRTIs). NRTIs form the backbones of most first- and second-line antiretroviral therapy (ART) regimens formulated for the treatment of HIV-1 infection.

The activity of NRTIs depends on two factors: (1) the intracellular concentration of their triphosphorylated moiety since NRTIs require triphosphorylation by cellular kinases to act as competitors of the natural 2′ -deoxy-nucleoside trisphosphates (dNTPs); (2) the concentration of the cellular dNTP pools. MDM are resting cells, characterized by a limited DNA synthesis and consequently by a low intracellular level of dNTPs [11,30,31]. The resting status of MDM overcomes the low affinity of most NRTIs for kinases acting at their first phosphorylation step [11], and results in a competition by dNTPs lower in MDM than in CD4+ lymphocytes whose intracellular dNTPs pool is 6–20-fold greater than those found in MDM [33,34]. These metabolic characteristics may explain why all the NRTIs clinically available are more active in MDM than in CD4+ lymphocytes in *in vitro* biological models [11].

NNRTIs bind to a hydrophobic pocket adjacent to the active site of reverse transcriptase and cause a conformational change that reduces the ability of nucleosides to be added to the growing DNA chain. In general, the NNRTIs have a lower incidence of adverse effects in comparison to the NRTIs. When they were used as single agent, the drugs quickly suppress viral replication, but resistance and virologic relapse soon develop [35]. With the exception of etravirine and rilpivirine, resistance develops with the substitution of a single amino acid at the NNRTI binding site.

Since the NNRTI activity is not affected by the dNTP pools, substantial differences in the antiviral activity of NNRTI have not been observed between MDM and CD4+ lymphocytes [11]. Previous studies confirm these findings, the anti-HIV-1 activity of NNRTIs is not modulated by the macrophage colony-stimulating factor, which increases the dNTPs pool in MDM and thus capable to affect the activity of NRTIs [33].

Doravirine (DOR), a novel NNRTI, is active against HIV-1 and the most common NNRTI-resistant variants, and has a favorable, unique, and safety in vitro resistance profile in clinical trials. Because it is not a metabolic inducer or inhibitor [36,37], DOR is well absorbed, exhibits moderate protein binding activity and is not a perpetrator of pharmacokinetic drug–drug interactions. No clinically meaningful interactions were observed when DOR was co-administered with atorvastatin, oral contraceptives, magnesium-based antacids, or proton-pump inhibitors in healthy volunteers [36,38–40]. As a substrate of cytochrome P450 (CYP)3A4, exposure to DOR is reduced in the presence of moderate or strong inducers of CYP3A4 [36]. It has an elimination half-life of 12–21 h. DOR can be taken once daily without regard to food [36,41], and its bioavailability is not affected by age, gender, or moderate hepatic impairment [36,42,43]. Drug interaction studies have shown doravirine does not affect the pharmacokinetics of dolutegravir but may have its pharmacokinetics altered by rifampicin (rifampin) and other rifamycins (CYP3A inducers). No clinically significant interactions were noted between doravirine and pantoprazole, ledipasvir/sofosbuvir, or elbasvir/grazoprevir.

In previous studies, in a phase 2b study in treatment-naive adults, DOR at 100 mg QD with emtricitabine (FTC) and tenofovir disoproxil fumarate (TDF) demonstrated comparable efficacy to efavirenz with FTC/TDF and had a favorable safety profile, with lower rates of drug-related adverse events and CNS events than efavirenz [36]. In the phase 3 DRIVE-FORWARD trial, DOR at 100 mg QD demonstrated non-inferior efficacy and a superior lipid profile compared with darunavir-ritonavir after 48 weeks of combination treatment with 2 NRTIs [44].

Other researches are needed to better understand doravirine’s efficacy and safety profile when co-administered with other agents known to be CYP inducers or inhibitors.

Also, previous studies postulated that sterile alpha motif- and histidine/aspartic acid domain-containing protein 1 (SAMHD1) limits HIV-1 replication by hydrolyzing deoxynucleoside triphosphates (dNTPs) necessary for reverse transcription [45]. This report has shown that SAMHD1 cleaves NRTI triphosphates (TPs) at significantly lower rates than dNTPs and that SAMHD1 depletion from monocytic cells affects the susceptibility of HIV-1 infections to NRTIs in complex ways that depend not only on the relative changes in dNTP and NRTI-TP. The presence of SAMHD1, or its depletion, as occurs for lentiviruses that encode the Vpx accessory protein [46], can affect NRTI susceptibility in multiple ways that depend not only on the relative changes in the concentrations of dNTPs and NRTI-TPs but also on the activation pathways of NRTIs. This work highlights the importance of the metabolic pathways for activation of different NRTIs to NRTI-TPs, especially in cells in which dNTP concentrations are low and competition with NRTI-TPs does not mask the effects of differential NRTI activation.

SAMHD1 is the dominant host factor controlling post‐entry permissivity to infection of non‐dividing MDM. In recent works, it was demonstrated that SAMHD1 phosphorylation status at T592 controlling antiviral activity is naturally dynamic in primary human MDM. Also, the dynamic phosphorylation of SAMHD1 by CDK1 is associated with the expression of proteins typically associated with cell cycle control. Mlcochova et al. have shown that MDM can be stimulated to exit G0 phase and enter a G1‐like state. This transition with associated CDK1 upregulation and T592 phosphorylation of SAMHD1 renders macrophages permissive to HIV‐1 [47].

Mlcochova et al. have proposed a mechanism where ETO‐induced DNA damage induces SAMHD1 dephosphorylation via a canonical p53 and p21 pathway in macrophages. As a consequence of this regulation, activated dephosphorylated SAMHD1 mediates a block to HIV‐1 nuclear import and integration in MDM [48]. Therefore, topoisomerase inhibitors regulate SAMHD1 and HIV permissivity at a post-RT step, revealing a mechanism by which the HIV-1 reservoir may be limited by chemotherapeutic drugs [48].

Jáuregui and Landau postulated that the Growth of MDMs under conditions that alter the cell cycle alters SAMHD1 phosphorylation and affects their susceptibility to infection by HIV-1. *In vivo*, MDMs are differentiated as M1, M2. Because MDMs play a role in HIV-1 replication, alterations in SAMHD1 restriction activity could influence HIV-1 replication. While DNA damage agents are not likely to be useful in the treatment of HIV-1 infection, it may be possible to control the SAMHD1 phosphorylation state by targeting intracellular signal transduction in MDMs, thereby reducing virus loads [49].

## HIV-protease inhibitors

The PI bind competitively to the substrate site of the viral protease, responsible for the post-translational processing and cleavage of a gag and gag-pol polyprotein during budding from the infected cell. Their potent anti-HIV activity and introduction in clinical use in 1996 was one of the main reasons for the observed substantial falls in morbidity and mortality associated with HIV infection in the developed world [50].

HIV PIs prevent cleavage of gag and gag–pol poly-protein in acutely and chronically infected cells, arresting maturation and thereby blocking the infectivity of nascent virions [51]. PIs act during maturation of viral particles in the late stage of HIV-1 life cycle, thus, these drugs can inhibit the release of infectious viral particles from an already infected cell thus preventing subsequent waves of infection. The difference in the anti-HIV-1 activity of PIs in HIV-1 chronically infected MDM and CD4⫹ T lymphocytes may be explained by the high and sustained RNA metabolism in MDM, which affords a great production of virus particles, even from a limited amount of proviral DNA in these cells. Consistent with this hypothesis, HIV-RNA production from chronically infected MDM is not at all affected by PIs, even when protein maturation and release of infectious virus particles are inhibited signiﬁcantly [7]. This may have important clinical implications. In fact, the high concentration of PIs required to suppress HIV-1 replication in chronically infected MDM is often higher than through the PI concentration in plasma of treated patients.

## Integrase inhibitors

The first INIs were reported approximately 20 years ago [52]. Approximately 40–100 integrase molecules are packaged within each HIV particle. The primary role of integrase is to catalyze the insertion of the viral cDNA into the genome of infected cells, although integrase can also act as a cofactor for reverse transcription [52–54]. Integration is required for viral replication, because transcription of the viral genome and the production of viral proteins requires that the viral cDNA is fully integrated into a chromosome [52]. Following reverse transcription, the viral cDNA is primed for integration by integrase-mediated trimming of the 3′-ends of the viral DNA [52]. This step is referred to as 3′-PROCESSING. It requires both fully functional integrase and the integrity of the last 10–20 base pairs at both ends of the viral cDNA. 3′-processing consists of the endonucleolytic cleavage of the 3′-ends of the viral DNA. This cleavage occurs immediately 3′ to a conserved CA dinucleotide motif. Alterations of this sequence prevent integrase from catalyzing 3′-processing [52].

There are totally three integrase inhibitors [raltegravir (RAL); elvitegravir (EVG); dolutegravir (DTG)], which have been approved by FDA.

Scopelliti and colleagues have shown that HIV replication is inhibited by INIs in MDM at similar, or even lower, concentrations than those active in PBMCs. This supports the hypothesis that RAL (and other INIs) are able to control the spreading of HIV in MDM and in actively replicating T cells [55].

Pollicita et al. have shown that MDMs and PBMCs might act as reservoirs more for wild-type virus than for resistant/low-fitness virus due to a measurable advantage in the replication capacity of wild-type virus compared with RAL-resistant strains in all tested cell systems. Dolutegravir efficiently reduces HIV-1 replication in MDMs, PBMCs, and C8166 cells, with the potential to be effective in different HIV cellular targets, and against RAL-resistant strains harboring the Y143 and N155 mutations [56].

Courtney et al. have found that drug penetration for many commonly used ARVs was lower in lymphoid tissue cells than that observed in blood cells. Indeed, in this study, it was shown that measures of virus replication in blood do not necessarily reflect the impact of ARVs on virus production at its principal source in lymphatic compartments. These findings support the hypothesis that ARV concentrations in lymphatic tissue can be insufficient to fully suppress HIV-1 replication and that measuring VL in PB will not necessarily reflect virus production at its source in tissues.

## BIC (Bictegravir)

In the recent work of Smith and col., based on their antiviral analysis of the ability of these new INIs to inhibit previously identified INIs-resistant single, double, and triple mutants in a single-round replication assay, it appears that BIC is more broadly effective than either of the first-generation INIs, RAL and EVG. In terms of their ability to inhibit INIs-resistant mutants that were tested, BIC was significantly better than DTG [32]. These conclusions concerning the relative efficacies BIC against mutants are in good agreement with the data of other studies like Yoshinaga et al. [57] and Neogi et al. [58]. Based on these results, BIC appears to be a very promising INIs. Nonetheless, based on experience with previous ARV drugs, in the long term, resistant viruses will emerge.

## Entry inhibitors

The envelope glycoprotein complex (Env) is responsible for the entry of HIV-1 into cells by mediating attachment to target cells and subsequent membrane fusion. Env consists of three gp120 subunits that mediate receptor and co-receptor attachment and three gp41 subunits responsible for membrane fusion. Several steps of the entry process can serve as drug targets. CCR5 antagonists prevent attachment of gp120 to the CCR5 or co-receptor and conformational changes within gp41 required for membrane fusion can be inhibited by fusion inhibitors. The fusion inhibitors (FIs) target the HIV-1 glycoprotein gp41, thus preventing the fusion between the viral and the host cell membrane. Representative fusion inhibitors include: the first FDA-approved HIV-1 fusion inhibitor T20 (generic name: enfuvirtide; brand name: Fuzeon) [44,59], C34, T1249 [60,61], T2635 [62] and sifuvirtide [63]. It has been demonstrated by using different lab-adapted HIV-1 strains, that T-20 may efﬁciently prevent the entry of HIV-1 into PBMC, MDM, and immature DC [64]. It is interesting to note that the T-20 susceptibility may be modulated by coreceptor speciﬁcity [65]. In particular, it has been demonstrated that CCR5-using strains are characterized by an intrinsic resistance to T-20, and thus, their replication is suppressed at concentrations of T-20, higher than those required for CXCR4-using strains [65]. Moreover, the clinical efﬁcacy of T-20 is also strengthened by the fact that the development of drug resistance [66–69] may be associated with immunological success, despite virological failure [70].

A new class of HIV entry inhibitors, CD4 attachment inhibitors, targets the first step of entry. Fostemsavir (FTR) is a small molecule inhibitor that binds HIV envelope glycoprotein 120 (gp120) and prevents virus from binding to the CD4 receptor. It inhibits the entry of HIV into CD4 + T-lymphocytes by blocking conformational changes in gp120. This mechanism of inhibition is different from that of other entry inhibitors targeting co-receptor binding (maraviroc) or fusion (enfuvirtide). FTR is an oral prodrug of temsavir, the active compound, with potent activity in vitro against HIV, and pharmacokinetics that support once-daily dosing with no need for pharmacologic boosting. Initial clinical investigations have revealed the safety and efficacy of 8-day monotherapy with fostemsavir in HIV-infected individuals [71,72]. A Phase IIb study has shown safety, tolerability, and equivalent efficacy of cART including fostemsavir in comparison with a cART including ritonavir-boosted atazanavir in a randomized active-controlled trial [71,73,74].

The chemokine receptor CCR5 (belonging to the family of the seven-transmembrane domain proteins) is expressed by MDM and represents the most important coreceptor for M-tropic HIV-1 strains to enter the cells. CCR5 plays a crucial role in the transmission of HIV-1 isolates that establish initial infection, persist during the early years of infection, and predominate in brain where HIV causes neuro-AIDS [73]. Previous studies have shown that TAK-779 is the first non-peptidic molecule known to block selectively the replication of CCR5-using HIV strains in MDM at low concentrations (about 10 nM). TAK779 interacts directly with CCR5 in a specific binding site within the transmembrane domain helices 1, 2, 3, and 7. TAK-779 has a high affinity for CCR5, and totally inactive against CXCR4-using strains of HIV-1 [73,75].

Maraviroc is an allosteric modulator of CCR5 that stabilizes CCR5 in a conformation to which HIV Env does not bind [76–79]. Maraviroc treatment rarely causes a tropism switch to X4 viral strains in the absence of preexisting X4 strains [79]. Rather, some cases of maraviroc resistance in R5 HIV strains were reported in vitro and in treated patients. In these instances, HIV evolved the capacity to enter target cells by using the maraviroc-bound form of CCR5 as a coreceptor [80–82]. Recent clinical trials included maraviroc in an intensified regimen consisting of four or five antiretroviral drugs, with the objective of limiting the establishment of the viral reservoir in acute HIV infection, or of decreasing residual viral replication in chronic HIV infection [83–88]. Results of such trials have generally been disappointing, as intensified regimen did not prove superiority compared to classic regimens in containing the viral load or in decreasing the HIV reservoir. Unfavorable drug interactions may have limited the benefit of therapy intensification in some cases [89]. A paradoxical increase in T-cell activation was reported in one trial, possibly due to an increase in the concentration of circulating chemokines [84]. However, in another trial, maraviroc intensification was associated with an improvement of the CD4 recovery slope in patients with poor immunological restoration [83]. Surdo M et al., 2013 have shown Maraviroc is effective in-vitro against viruses with dual-characteristics in both MDM and lymphocytes, despite the potential X4-mediated escape [90]. This suggests that the concept of HIV-entry through one of the two coreceptors “separately” may require revision, and that the use of CCR5 antagonists in patients with dual/mixed-tropic viruses may be a therapeutic option that deserves further investigations in different clinical settings.

## Towards new therapeutic strategies: maturation inhibitors

Developing inhibitors against novel targets provides a wealth of basic mechanistic information about fundamental aspects of viral replication. A new class of inhibitors is now emerging that targets the internal structural precursor protein, Gag, and its function in the final assembly of the mature, infectious virion, these are the maturation inhibitors (MI). This new class is typified by the compound 3-O-(3ʹ,3ʹ-dimethylsuccinyl)-betulinic acid, known alternatively as Bevirimat (BEV) [91–93], PA-457 [92] or DSB [93]. In this regard, the maturation of HIV-1 particles, which is triggered by the action of the viral PR, occurs concomitantly with virion release from the infected cell [94–96]. PR cleaves a number of sites in the Gag polyprotein precursor, Pr55Gag, the major structural protein responsible for the formation of virus particles. PR-mediated Gag cleavage gives rise to the matrix (MA), capsid (CA), nucleocapsid (NC), and p6 proteins and to two small spacer peptides, SP1 and SP2, located between CA and NC and between NC and p6, respectively.

Initially there is rapid cleavage at the C-terminus of SP1, followed by cleavage between MA and CA. Additional cleavage events then occur, with the final (slowest) reaction separating SP1 from the C-terminus of CA [97]. This final cleavage event is critical in proper virion morphogenesis, as disruption of this cleavage site through mutagenesis leads to aberrant core formation and the generation of noninfectious particles. In the immature particle, the Gag precursor proteins are arranged radially around the outer edge of the virus particle, whereas in the mature virion the CA proteins assemble into a centrally located, conical core (referred to as the capsid) in which the viral RNA genome and the viral enzymes RT and IN reside [97,98]. Each processing site within the Gag and Gag-Pol polyprotein precursors is cleaved by PR with distinct kinetics, largely due to the unique primary amino acid sequence at each site [96]. The consequence of the differential rates of cleavage is that Gag and Gag-Pol processing occurs as a highly ordered cascade of cleavage events. This highly ordered processing is required for proper maturation. Defects in maturation can affect both virus entry [96,99] and subsequent postentry events. Even partial disruption of processing at several sites in Gag leads to severely impaired virus infectivity [96,100–102], highlighting the utility of Gag processing as a target for antiretrovirals.

BEV, the first HIV-1 MI, was identified through analyzing natural products coupled with an activity-directed structural modification effort [92,93,103]. BEV, also called PA-457, is a derivative of betulinic acid and has potent antiviral activity against multiple wild-type and drug-resistant clinical HIV-1 isolates. Despite potent activity against HIV-1, this antiretroviral drug is inactive against HIV-2. BEV disrupts a late step in Gag processing involving conversion of the CA precursor (CA-SP1) to mature CA [92,93,103]. Virions from BEV-treated cultures are noninfectious and exhibit an aberrant particle morphology characterized by a spherical, acentric core and a crescent-shaped, electron-dense shell lying just inside the viral membrane. Although BEV specifically disrupts CA-SP1 cleavage, it has been shown that the compound does not affect the viral PR function [92,93,103]. Moreover, consistent with the effect on Gag processing, the determinants of BEV activity map to amino acid residues flanking the Gag CA-SP1 cleavage site [92,93,103–107]. BEV represents a novel class of anti-HIV compounds termed MIs that exploit a previously unidentified viral target.

In 2009, results from Study 204, a phase II dose-ranging clinical trial, were presented. While not showing impressive antiviral activity, a genotypic test has been developed to predict those most likely to respond to the drug. Later, BEV was renamed bevirimat MPC-4326 and also is developing HIV maturation inhibitors of its own, which are code-named MPC-9055 and MPC-461359. Of these three drugs, MPC-4326 (bevirimat dimeglumine) is the furthest along in clinical development. MPC-4326 was originally developed in a liquid formulation. However, a tablet formulation is now used in clinical trials.

Results from previous clinical trials of MPC-4326 suggest that the drug is generally well tolerated. Headache is the most common side effect and even this was mild in severity. Many anti-HIV drugs are broken down in the liver or kidneys by different enzymes. An advantage of MPC-4326 is that it is not processed by the more common pathways in the liver that often lead to significant drug–drug interactions. MPC-4326 is processed in the liver by the same class of enzymes (called UGTs) that metabolize some other anti-HIV drugs such as RAL (Isentress). Very small portion of MPC-4326 is processed by the kidneys. This means there are few other HIV drugs that will change the way MPC-4326 is processed in the body. Additionally, laboratory studies suggest that MPC-4326 does not inhibit the liver enzyme cytochrome P450 3A4 which processes many anti-HIV drugs. Therefore, there is a low likelihood for MPC-4326 to affect the processing of other anti-HIV drugs and consequently have little impact on the levels of other anti-HIV drugs in the body.

Mechanism-of-action studies were conducted to ensure that inhibition of maturation was maintained as the inhibitory mechanism. This process led iteratively to the identification of BMS-955176, which contains major structure-activity-directed modifications to elements peripheral to the betulinic acid core structure, as well as to the core itself, compared to BVM. BMS-955176 is a second-generation MI that also inhibits this single, specific HIV-1 *PR* cleavage event between CA and SP1 in *Gag*, producing immature, noninfectious virus particles. However, it exhibits potent activity toward the polymorphic variations in Gag associated with resistance to BVM [108–110].

In a previous work, Nowicka-Sans et al. 2016 identified and characterized BMS-955176, a second-generation HIV-1 maturation inhibitor with improved potency, antiviral spectrum, and polymorphic coverage [108]. In vitro combination studies showed that BMS-955176 MI has a potent in vitro anti-HIV-1 activity and a greatly improved preclinical profile compared to that of bevirimat [108].

In our laboratory, projects related to the test of the antiviral capacity of the MI are being carried out. There are ongoing trials in which the antiviral activity of these inhibitors in vitro in macrophages is tested. The research proceeds successfully but still requires more experiments and studies since the results obtained are good but very preliminary.

## Recent strategies and challenges facing delivery of antiretroviral therapies

Sadowski and Hashemi have observed that the development of a routine effective cure will inevitably require the eradication of these latently infected reservoirs [111]. Latently infected cells theoretically would be devoid of viral proteins and antigenically indistinguishable from non-infected cells, but some evidence suggests that most latently infected cells produce sporadic occasional viral transcripts, through stochastic mechanisms that may maintain low levels of gene products that could produce a unique cellular identity, either directly or indirectly by affecting the expression of host cell proteins [112]. Targeting cell receptors on HIV-infected macrophages is an important challenge to the feasibility of most curative strategies for HIV/AIDS. As evidenced by the lack of progress in HIV vaccine development, targeting HIV proteins has been difficult. Host cell surface receptors are more readily targeted regardless of HIV infection status. The major roadblock for targeting macrophages is the limited availability of targeting ligands. Most potential targeting ligands are small-molecule chemicals whereby conjugation to a drug delivery carrier tends to reduce the ability of the ligand to bind to a target cell receptor. Little has been published in this area in general and specifically as it relates to HIV/AIDS. A priority must be put on the discovery of new cell-targeting ligands for potential nanocarriers, their optimal display on drug-loaded nanocarriers.

Lately, only a few potential cell targets on macrophages have been exploited due to concerns about the potential inference with host immune function or the lack of suitable ligands that can be conjugated to nanocarriers. Most targeting ligands have been peptides as the peptidyl nature makes it amenable to conjugation to a nanocarrier [113].

Several and important progress has been made in using human cells as drug delivery depot sites. These approaches have utilized macrophages as drug carriers. HIV drugs were first loaded into nanocarriers [114] or red blood ghost cells [115] that become engulfed by macrophages *ex vivo* or *in vivo*. The macrophages then migrated to lymph nodes or additionally to other reticuloendothelial system tissues, acting as a cellular depot in these critical sites of HIV infection where the drugs slowly diffuse out over a period of days to weeks resulting in sustained high local drug concentrations in the lymph nodes or other reticuloendothelial system tissue. In a feline immunodeficiency virus model [116], the membrane-impermeable HIV drug zalcitabine-TP was first loaded *ex vivo* into autologous red blood cells and the plasma membrane of these red blood cells was then chemically modified so macrophages would recognize and engulf them. In a 7-month experimental feline immunodeficiency virus infection, zalcitabine-loaded erythrocytes protected the majority of peritoneal macrophages and reduced the infection of circulating lymphocytes. The Gendelman group has developed long-acting/extended release nanoformulations of ritonavir, indinavir, and efavirenz (nanoART) [114]. They reasoned that circulating macrophages traveling across the blood-brain barrier could enhance nanoART brain delivery [114]. Mechanistic studies [117] showed that nanoART–macrophage interactions enhanced phagocytosis, secretory functions, and cell migration, which could be exploited to increase macrophage nanoART loading capacity. Aouadi et al. [118] studied anti-inflammatory small interfering RNA (siRNA) delivery utilizing yeast ghost cells. Yeast ghost cells were made by chemical treatment of yeast cell wall so that the cell surface was left with only beta1 3-D-glucan, for which macrophages have a special receptor. siRNA was then loaded into the ghost cells. The ghost cells can be efficiently absorbed orally through M-cells and, once crossed M-cells, avidly phagocytosed by macrophages in the Peyer’s Patches. Interestingly, macrophages in the Peyer’s Patches were able to migrate into blood circulation and settle at various LNs. Oral gavage of mice with the siRNA-loaded ghost cells containing as little as 20 μg/kg of siRNA directed against tumor necrosis factor alpha (TNF-α) depleted its messenger RNA in macrophages recovered from the peritoneum, spleen, liver and lung, and lowered serum TNF-α levels. Although not developed an HIV application, this approach is readily translatable to the delivery siRNA against HIV infection.

Also, other recent studies showed that a small population of human macrophages survive acute HIV infection and that these surviving infected cells become latently infected, as viral replication is not detected [119]. These the surviving cells exhibit metabolic compromise and mitochondrial fusion, lose reliance on Mitochondrial Oxidative Phosphorylation System, and accumulate lipids. Most of these changes could be mimicked by adding succinate or glutamine/glutamate to the cells, supporting a compromised tricarboxylic acid cycle. Despite these changes, no overall alterations in mitochondrial membrane potential or electron transport chain mitochondrial expression were detected. Besides, it was shown that while uninfected macrophages exclusively use glucose and fatty acids as major sources of energy, latently HIV-infected macrophages use glutamine/glutamate as a significant source and gain the capability to shift from one metabolic source to another. Furthermore, blocking the use of glutamate, glutamine, or α-KG results in the specific elimination of HIV surviving reservoirs. Together, these data identify a unique metabolic signature of latently infected cells (not replicated by immune activation), which could be pharmacologically targeted to eliminate HIV reservoirs [120].

## Conclusions

Several researches leading to the discovery and progress of HIV inhibitors that are now approved as anti-AIDS drugs are a unique source of information that could, in the future, be used in other attempts of structure-based drug design. Undoubtedly, structural studies are only one part of this complicated procedure. Specific assays of a large ensemble of crystal structures (such as, for example, the HIV PR database) can provide a surprising perspective to the problem of drug–target interactions and lead to the design of more efficient drugs. The coupling of such data with other in vitro and in vivo studies makes it possible to enhance the process of design.

The RTIs and the protease inhibitors are able to inhibit HIV replication in MDM but the limited development of drug resistance suggests that these drugs may efficiently suppress HIV replication in MDM (the main cellular reservoir of HIV infection) for a long time.

Moreover, the crucial role of MDM as a reservoir in the pathogenesis of HIV infection, especially in the CNS, underlines the importance of testing in MDM the antiviral efficacy of new drugs designed to target different stages of the HIV lifecycle. Particular attention has been dedicated to drugs able to target the CCR5 coreceptor selectively, the main coreceptor used by HIV to enter MDM. The CCR5 antagonists also represent a promising approach for their ability to synergize with T‐20, the first fusion inhibitor in clinical use [121] even under viral suppression by antiretroviral therapy.

Taken together, overall findings support the clinical relevance of interfering with HIV replication in MDM. In particular, the inherent properties of HIV infection of MDM should be taken into account in designing therapeutic strategies aimed at achieving an optimal, therapeutic effect in all tissue compartments where the virus hides and replicates. MIs are a new class of HIV drugs with an attractive clinical development profile [103]. Clinical proof-of-concept demonstrated by the first-in-class MI BEV and reappearance of second-generation drug candidates with improved breadth and potency in clinical trials strongly suggest that in the near future multiple MIs can be approved and used for the treatment of HIV/AIDS patients.
